# Revealing the complete mitochondrial genome of *Thermophis baileyi* Wall, 1907 (Reptilia: Colubridae) through the next-generation sequencing

**DOI:** 10.1080/23802359.2017.1347902

**Published:** 2017-07-07

**Authors:** Feng-Hui Sun

**Affiliations:** a Engineering Laboratory of Prevention and Control of Veterinary Drug Residues in Animal Derived Food, Chengdu Medical College, Chengdu, China;; b Chengdu Medical College, Chengdu, China

**Keywords:** Mitogenome, next-generation sequencing, thermophis baileyi

## Abstract

The complete mitochondrial genome of *Thermophis baileyi* was sequenced using the next-generation sequencing (NGS) in the present study. The total length of the mitogenome was 17,355 bp, which was composed of 13 protein-coding genes, 22 transfer RNA (tRNA) genes, 2 ribosomal RNA (rRNA) genes (12s and 16s rRNA), and 2 control regions (CR I and CR II). The base composition was 32.4% for A, 23.8% for T, 30.2% for C, and 13.6% for G. Coding genes of each protein in the mtDNA had the same start and stop codons among three *Thermophis* species.

Genus *Thermophis* contains only three species (*Thermophis zhaoermii*, *Thermophis shangrila*, *Thermophis baileyi*), which is distributed in a few sites at high altitudes (over 3500 m) (Huang et al. 2009; He et al. 2010; Alex et al. 2016). *Thermophis baileyi* is endemic to the Tibetan Plateau and was firstly described by Wall (1907) based on characters (Zhao 2008; Hofmann 2012). In this study, we determined the complete mitochondrial genome of *T. baileyi* using the next-generation sequencing (NGS).

Sample was collected from the Yangbajing, Xizang Privonce, China. Specimen was stored in the CIB herpetological museum, Chengdu Institute of Biology with the number of CIBLJT20150701. The genomic DNA isolated from the sample was sequenced using NGS (Hahn et al. 2013). We predicted and analyzed the mitogenomic structure using MITOS web server (Bernt et al. 2013). RefSeqs which were downloaded from NCBI (https://www.ncbi.nlm.nih.gov/genome/organelle/) was used to correct the mitogenomic structure manually. The tRNA genes were scanned by tRNAscan-SE (Lowe and Eddy 1997). MEGA5 was used to calculate the base composition and to align the whole-genome sequence data (Tamura et al. 2001). Maximum likelihood (ML) method was used to reconstruct a phylogenetic tree in the present study by using MEGA5 (Tamura et al. 2001).

The mitogenome of *T. baileyi* (GenBank MF326642) was 17,355 bp in length, with a base composition of 32.4% for A, 23.8% for T, 30.2% for C, and 13.6% for G. The mitogenome sequenced by us contained 13 protein-coding genes, 2 rRNA genes (12s and 16s rRNA), 22 tRNA genes, and 2 control regions (CR I and CR II). The ND 6 gene encoded on the light-strand, with the remaining protein coding genes encoding on the heavy strand. Compared with the other two hot-spring snakes (*Thermophis zhaoermii* NC012816; *Thermophis shangrila* MF066951), each protein-coding gene in the mtDNA had the same start and stop codon among three *Thermophis* species (He et al. 2010; Wu et al. 2017). The length of CRI was 1129 bp, ranging from 16,226 to 17,355 bp and CRII was 1027 bp, ranging from 3629 to 4655 bp. The length of 22 tRNA genes ranged from 57 bp to 73 bp.

A ML tree based on the total mitochondrial genome sequences from 26 species of snakes supported that *Thermophis baileyi*, *Thermophis zhaoermii* and *Thermophis shangrila* form a molophylitic clade (Figure 1). The evolutionary relationships of these species were consistent with previously reported results (Alex et al. 2016). The mitogenome characterization of the *T. baileyi* was helpful to understand the evolution of Colubridae.

**Figure 1.**
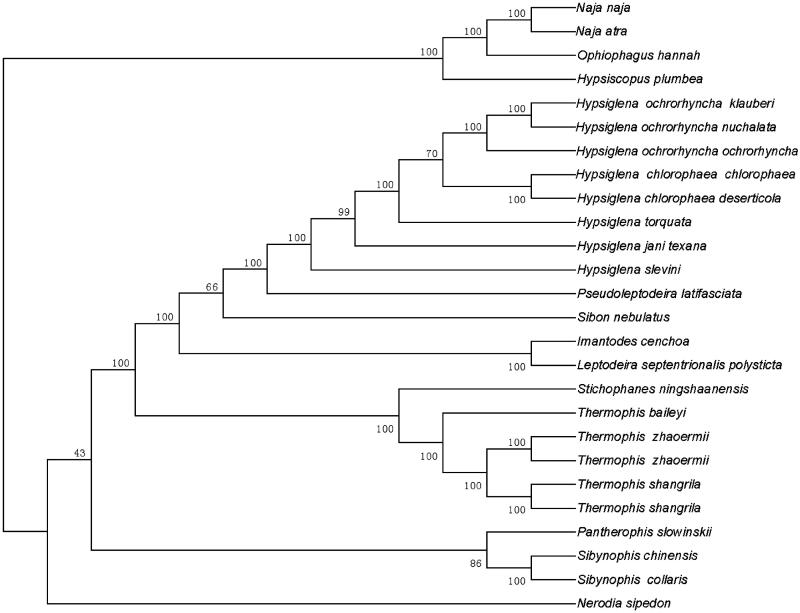
Molecular phylogeny of *Thermophis baileyi* and other related species in ferns based on complete mitochondrial genome. The sequences accession number for tree re-construction is listed as follows: *Thermophis baileyi* (MF326642); *Hypsiscopus plumbea* (DQ343650); *Nerodia sipedon* (JF964960); *Pantherophis slowinskii* (DQ523162); *Sibynophis chinensis* (NC022430); *Sibynophis collaris* (NC016424); *Thermophis zhaoermii* (GQ166168); *Thermophis zhaoermii* (NC012816); *Thermophis shangrila* (MF066951); *Thermophis shangrila* (KU174488); *Pseudoleptodeira latifasciata* (EU728579); *Hypsiglena chlorophaea chlorophaea* (EU728593); *Hypsiglena chlorophaea deserticola* (EU728587); *Hypsiglena jani texana* (EU728592); *Hypsiglena ochrorhyncha klauberi* (EU728589); *Hypsiglena ochrorhyncha nuchalata* (EU728581); *Hypsiglena ochrorhyncha ochrorhyncha* (EU728578); *Hypsiglena slevini* (NC013987); *Hypsiglena torquata* (EU728591); *Imantodes cenchoa* (EU728586); *Sibon nebulatus* (EU728583); *Leptodeira septentrionalis polysticta* (EU728590); *Stichophanes ningshaanensis* (NC026083); *Naja atra* (EU913475); *Ophiophagus hannah* (NC011394); *Naja naja* (DQ343648).
